# Effect of intranasal and oral administration of levetiracetam on the temporal and spatial distributions of SV2A in the KA‐induced rat model of SE

**DOI:** 10.1111/jcmm.17986

**Published:** 2023-10-16

**Authors:** Weixuan Zhao, Yue Li, Huaiyu Sun, Wuqiong Zhang, Jiaai Li, Ting Jiang, Li Jiang, Hongmei Meng

**Affiliations:** ^1^ Department of Neurology, The First Hospital of Jilin University Jilin University Changchun China; ^2^ Department of Neurology, Qingdao Women and Children's Hospital of Qingdao University Qingdao University Qingdao China; ^3^ Department of Neurology The First People's Hospital of Lishu County Siping China

**Keywords:** epilepsy, hippocampus, intra‐amygdala kainic acid model, levetiracetam, nasal drug delivery, SV2A

## Abstract

To investigate the effectiveness of nasal delivery of levetiracetam (LEV) on the distributions of synaptic vesicle protein 2 isoform A (SV2A) in epileptic rats with injection of kainic acid (KA) into amygdala. A total of 138 rats were randomly divided into four groups, including the Sham surgery group, the epilepsy group (EP), and the LEV oral administration (LPO) and nasal delivery (LND) groups. The rat intra‐amygdala KA model of epilepsy was constructed. Pathological changes of rat brain tissue after status epilepticus (SE) were detected using haematoxylin and eosin staining. Expression of SV2A in rat hippocampus after SE was evaluated using the western blotting analysis. Expression and distribution of SV2A in rat hippocampus after SE were detected based on immunofluorescence staining. The EP group showed evident cell loss and tissue necrosis in the CA3 area of hippocampus, whereas the tissue damage in both LPO and LND groups was significantly reduced. Western blotting analysis showed that the expressions of SV2A in the hippocampus of both EP and LND groups were significantly decreased 1 week after SE, increased to the similar levels of the Sham group in 2 weeks, and continuously increased 4 weeks after SE to the level significantly higher than that of the Sham group. Results of immunofluorescence revealed largely the same expression patterns of SV2A in the CA3 area of hippocampus as those in the entire hippocampus. Our study revealed the same antiepileptic and neuronal protective effects by the nasal and oral administrations of LEV, without changing the expression level of SV2A.

## 1 INTRODUCTION

Epilepsy is a brain disease caused by abnormal firing of highly synchronized neurons in the brain.^1^ In general, multiple etiologies could lead to epileptic seizures. However, the complex pathogenesis of epilepsy has not been fully elucidated. Currently, epilepsy is considered the ‘ion channel disease’ with the pathogenesis closely related to the mutations of genes involved in the Na^+^, K^+^, Ca^2+^ and Cl^−^ channels.^2^ To date, the main clinical treatment of epilepsy is the prophylactic use of antiepileptic drugs (AEDs), which are used to control about two‐thirds of the epileptic seizures. However, some AEDs have shown severe side effects, for example, liver and kidney damages and allergic reactions as well as low oral bioavailability.^3^, ^4^, ^5^ For example, the carbamazepine is widely known as the preferred treatment for epilepsy. However, the oral therapy of carbamazepine results in slower brain uptake and systemic side effects. For example, Acharya et al. prepared the intranasal oil in water microemulsion of carbamazepine to improve its solubility and enhance the brain uptake, achieving significantly increased antiepileptic effect and higher uptake rate of the medicine in the brain tissue.^3^ Similarly, it has been proposed that a reduction of systemic bioavailability of levetiracetam (LEV) was demonstrated in patients co‐medicated with enzyme inducing AEDs (i.e. carbamazepine, phenytoin and phenobarbital), while patients treated with LEV also showed drug resistance due to the presence of BBB and blood‐cerebrospinal fluid barrier.^4^ In particular, a thermoreversible gel loaded with LEV was administered to CD‐1 male mice by intranasal route and the pharmacokinetics was compared to those observed after the intravenous administration. The similar plasma pharmacokinetic profiles were obtained and the intranasal absolute bioavailability was significantly increased.^4^ These results evidently suggest the advantages of nose‐to‐brain delivery of drugs, which was highly convenient and efficient.

As recently reported by the World Health Organization, the number of epilepsy cases has exceeded 50 million worldwide, with 10 million cases in China and more than 60% identified in children and teenagers.^6^ In particular, there are 500,000 new cases detected annually in China^7^ with ~25% diagnosed as refractory epilepsy (RE).^8^ The generalized RE is defined as the cases which have been clinically diagnosed as RE and epilepsy syndrome or with seizures which cannot be stopped by the standard treatment of the current AEDs.^9^ To date, the use of AEDs is still the main clinical treatment for RE. However, the blood–brain barrier (BBB) prevents almost 100% of macromolecular drugs and more than 98% of small‐molecule drugs from reaching the brain lesions to exert their therapeutic effects.^10^

**FIGURE 1:**
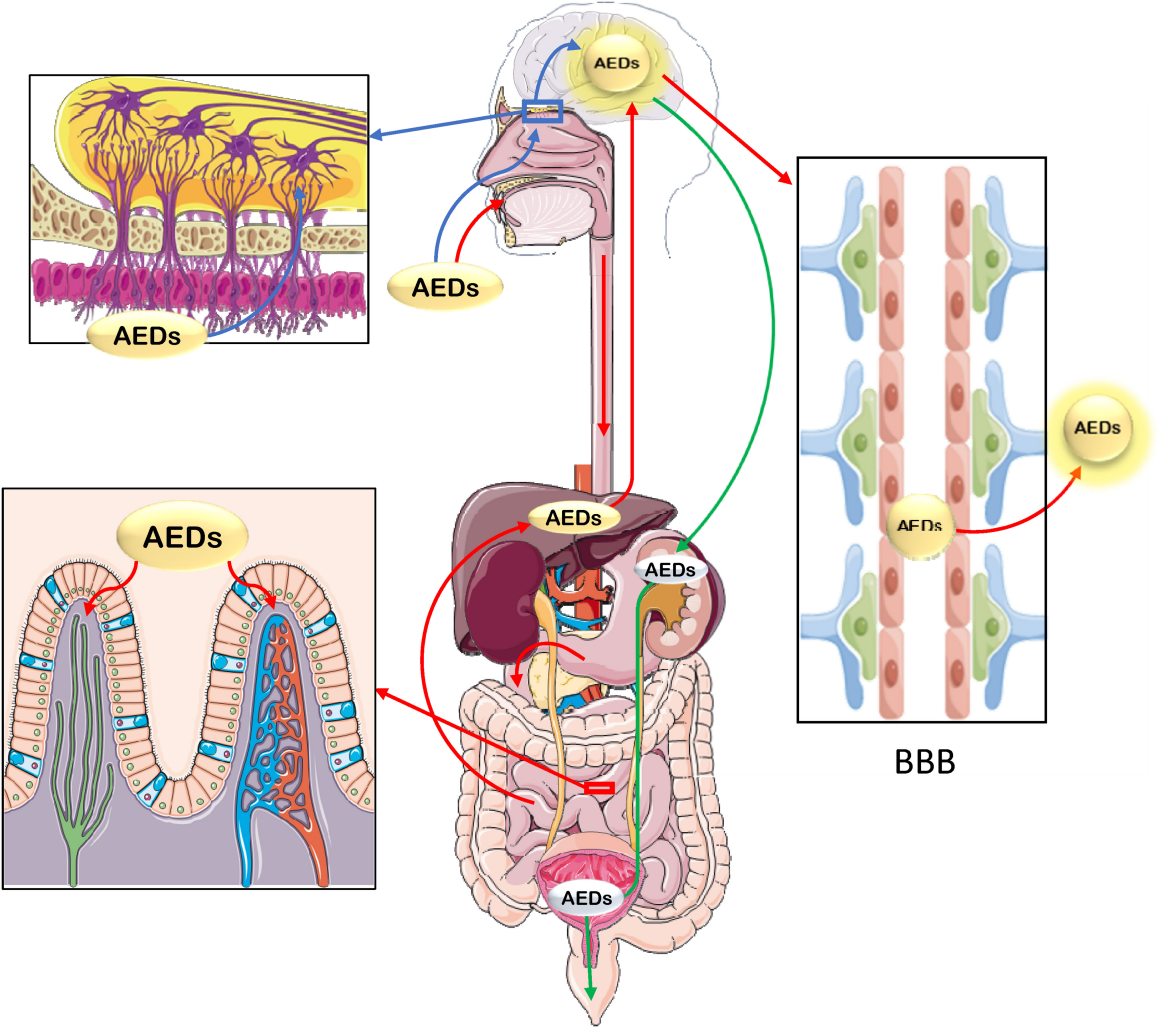
Schematic diagram of antiepileptic drugs (AEDs) entering the human body and their metabolic paths. The red lines indicate that the AEDs are taken orally, absorbed into the blood by the villous epithelial cells of the small intestine, metabolized by the liver, entering the brain through the BBB and reaching the epileptogenic focus to play their roles. The blue lines indicate that the AEDs are administered through the nasal cavity, entering the brain directly through the olfactory bulb and nasal mucosa and reaching the epileptogenic focus to play their roles. The green lines indicate that the AEDs enter the blood after exerting their effects in the brain, and then are excreted after being metabolized by the kidneys or directly out of the body.

Since 1986, a large number of animal experiments and clinical studies have revealed the unique connection between nasal mucosa and brain due to their physiological function and anatomy. For example, some drugs that could hardly pass through the BBB could reach the central nervous system via the nasal drug delivery (NDD).^11^ Furthermore, the NDD has shown the advantages of avoiding the liver first‐pass effect, reducing the systemic side effects of the entire body and high bioavailability as well as the fast drug absorption and rapid onset of function (Figure 1). Moreover, the absorption rates, plasma concentrations and pharmacokinetics of NDD are also superior to intravenous administration.^12^, ^13^

As one of the currently widely used AEDs, LEV has shown a wide range of applications in different populations with broad spectrum of functions, that is, significant curative effect on RE and protection of cognitive function in patients. It has been proposed that the molecular mechanism underlying the therapeutic effects of LEV is based on its specific binding to the synaptic vesicle protein 2 isoform A (SV2A) to inhibit the abnormal discharge of the epilepsy circuit, ultimately preventing the occurrence of seizures.^14^ Studies showed that as the important positive regulator of synaptic transmission in the brain, SV2A is involved in the regulation of the release, exocytosis and recycling of the vesicles, while the changes in the expression of SV2A make significant impacts on the effect of LEV.^15^

SV2A is generally considered functioning as a transporter.^16^, ^17^, ^18^ Studies showed that the SV2A‐deficient mice appeared normal at birth but began to develop generalized convulsions 6–10 days after birth, which was the time when GABA changed from excitatory to inhibitory transmitters in the development of the central nervous system. Mice with convulsive seizures due to SV2A‐deficient died within 3 weeks after birth.^19^ Kaminski et al.^20^ reported that the SV2A knockout (±) heterozygous mice were not revealed with any spontaneous seizures. However, a reduced seizure threshold of SV2A (±) mice was observed in models of pilocarpine, kainic acid (KA), pentylenetetrazol and 6‐Hz. The anticonvulsant efficacy of LEV, defined as the ability to increase seizure threshold for 6 Hz electrical stimulation, was significantly reduced (~50%) in the SV2A (±) mice, which was consistent with the reduced binding to SV2A in these mice. These results suggested that the partial loss of SV2A could lead to increased susceptibility to epilepsy.

The expression and distribution of SV2A during different stages of epilepsy are important for revealing the antiepileptic mechanism of LEV and the patterns of the occurrence and development of epilepsy, providing significant guidance for the clinical treatment and prevention of epilepsy. The goals of our study were to investigate the patterns of the spatial and temporal distributions of SV2A in the hippocampus of the rat intra‐amygdala KA model of epilepsy induced by the injection of KA and to further explore the effects of LEV administrated nasally on the expression and distribution of SV2A. Our results confirmed the antiepileptic effect of LEV and provided novel experimental evidence to support the further exploration of the molecular mechanisms regulating the functions of LEV and the temporal and spatial patterns of its therapeutic effect.

## 2 MATERIALS AND METHODS

### 2.1 Animals and chemicals

A total of 150 healthy adult male Wistar rats (280–300 g) were purchased from Yisi Laboratory Animal Technology Co., Ltd. The selection of male rats was to exclude the effect of estrous cycle of female rats on the experiments. The animals were uniformly raised in the Animal Room of the Department of Neurology, Translational Medical College, Jilin University, with the temperature maintained at 23 ± 3°C and free access to food and water. The levetiracetam (purity 99.9%) was obtained from Zhejiang Puluo Jiayuan Pharmaceutical Co., Ltd. The isoflurane was purchased from Tianjin Guangfu Institute of Fine Chemicals. The KA was purchased from Sigma‐Aldrich (Shanghai Trading Co., Ltd.).

A total of 42 rats were assigned into the Sham surgery group and each was given the same volume of PBS solution injected into the amygdala as that of the other groups of rats. The other 108 rats were used to construct the models of status epilepticus (SE) by the injection of KA into the amygdala. A total of 96 rats were successfully constructed as SE models and were randomly divided into three experimental groups (4 rats failed and 8 died), that is, the SE group (EP; 42 rats), the LEV group with LEV administrated orally (LPO; 12 rats), and the LEV group with LEV administrated nasally (LND; 42 rats). The four groups of rats were further treated in the following procedures: (1) Construction of the rat intra‐amygdala KA model of epilepsy. The rats in each of the Sham, EP, LPO and LND groups were sacrificed at 24 h, 3 days, 1 week, 2 weeks, and 4 weeks after the amygdala‐kindling surgery, respectively, to collect the brain tissues. Each subgroup contained a total of six animals at each time point. The rats in LND group were given 50 μL LEV (200 mg/mL; dissolved in normal saline solution) via nasal cavity 30 min prior to the surgery, then given the same amount of drug every 12 h until they were sacrificed. The rats in LPO group were given 1 mL LEV (25 mg/mL) by gavage 30 min prior to the surgery, then given the same amount of drug every 12 h until they were sacrificed. Both Sham and EP groups were given the same volume of normal saline by nasal cavity or gavage at the same time point of treatment until they were sacrificed. (2) Pathological changes of rat brain tissue after SE based on haematoxylin and eosin staining. The 12 rats in each of the Sham, EP, LPO and LND groups were sacrificed at 24 h and 3 days after the amygdala‐kindling surgery, respectively, to collect the brain tissues used to make the paraffin sections (5 μm thickness) for haematoxylin and eosin staining. Treatments of each group were the same as described above. (3) Expression of SV2A in rat hippocampus after SE based on the Western blotting analysis. The 18 rats in each of the Sham, EP and LND groups were sacrificed at 1, 2 and 4 weeks after the intra‐amygdala KA surgery, respectively, to collect the hippocampus tissue for western blotting analysis. Treatments of each group were the same as described above. (4) Expression and distribution of SV2A in rat hippocampus after SE based on immunofluorescence staining. The 12 rats in each of the Sham, EP and LND groups were sacrificed at 24 h, 1 weeks, 2 weeks and 4 weeks after the intra‐amygdala KA surgery, respectively, to collect the brain tissues used to make the paraffin sections (5 μm thickness) for immunofluorescence staining. Treatments of each group were the same as described above.

### 2.2 Rat intra‐amygdala KA model of epilepsy

The rats were anaesthetised by intraperitoneal injection of isoflurane (3% for induction and then 1.5% for maintenance). The head of the rat was fixed on the stereotaxic instrument with the horizontal scales of the two external auditory canals maintained at the same level. The incisor brackets were adjusted so that the anterior and posterior fontanelles of the rat were parallel to the desktop, that is, the head of the rat was kept horizontal. Then, the skin of the head of the rat was prepared and disinfected, and a longitudinal incision of 2–3 cm long was made along the middle of the head to separate the skin, fascia and other soft tissues to expose the coronal suture as well as the anterior and posterior fontanelles. An electric cranial drill for small animals was used to drill through the skull positioned at 2.5 mm behind the bregma and 4.5 mm on the right side to the surface of the dura mater. Then, the microinjector was fixed with the needle slowly inserted to the depth of 8.5 mm from the skull and 0.6 μg KA (in 0.6 μL of 1 μg/μL KA solution) slowly injected. The needle was maintained inside for 10 min after the injection and then slowly withdrawn. In the Sham group, the same amount of PBS buffer (0.6 μL) was slowly injected. The skin incision was aseptically sutured with the rat ear tag used to mark the animals undergone surgery. The rats were kept warm after the surgery until they were fully awake.

The successful construction of the rat model of SE was evaluated according to the Racine scales,^21^, ^22^, ^23^, ^24^ that is, grade 0: no behavioural changes; Grade 1: facial clonus, including blinking, whiskers and rhythmic chewing; Grade II: Grade I behaviours plus rhythmic nodding or tail flicking; Grade III: Grade II behaviours with myoclonus of the forelimbs but no hindlimb upright position; Grade IV: Grade III behaviours as well as the hindlimb erection; and Grade V: generalized tonic–clonic seizures and loss of postural control with fallings. The successful establishment of the rat model of SE was determined based on the occurrence of seizures described in grade IV or above for 1 h after the surgery. These seizures of grade IV and above were terminated by intraperitoneal injection of diazepam (8 mg/kg).

### 2.3 Haematoxylin and eosin staining of rat brain tissue

After deep anaesthesia, the experimental rats were injected with PBS buffer pre‐cooled at 4°C from the left ventricle, rapidly with 100 mL 4% paraformaldehyde pre‐cooled at 4°C, and then with 150 mL paraformaldehyde for fixation. The entire brain was immersed in 4% paraformaldehyde for 24 h for further fixation. With the needle track as the center, the brain tissue was trimmed into 6 mm thick blocks and washed with running water for 2 h, then dehydrated with ethanol gradient, cleared in xylene and immersed and embedded in wax for making sagittal cut of brain with continuous sections (5 μm thickness) mounted on glass slides.

### 2.4 Western blotting analysis of SV2A in rat hippocampus

Total hippocampal proteins were extracted, separated by polyacrylamide gel electrophoresis, and then transferred to polyvinylidene fluoride membranes. The membranes were blocked with 5% nonfat milk for 2 h at room temperature, washed three times with tris‐buffered saline Tween (TBS‐T) solution, mixed with primary antibody (rabbit SV2A antibody, Abcam, UK; 1:1000 dilution), and incubated overnight at 4°C. Then, the membrane was washed three times with TBS‐T and incubated with horseradish peroxidase‐conjugated secondary antibody (goat anti‐rabbit IgG antibody, Bioss, Shanghai, China; 1:10000 dilution) at room temperature for 90 min. The membrane was washed thrice with TBS‐T solution and the protein bands were detected using enhanced chemiluminescence (ECL) kit (Beyotime Biotechnology, Shanghai, China). Finally, the protein band intensities were quantified using ImageJ software.

### 2.5 Immunofluorescence staining of SV2A in rat hippocampus

Tissue sections of rat hippocampus were placed in 0.01 M citrate buffer for antigen retrieval, blocked with 5% BSA for 30 min, mixed with primary antibody (rabbit SV2A antibody and mouse GAD67 antibody, Abcam, UK; 1:100 dilution), and incubated at 4°C for 12 h in the dark. Then, the sections were washed three times with 0.01 M PBS buffer and incubated with secondary antibody (goat anti‐mouse IgG/Alexa Fluor594 and goat anti‐rabbit IgG/Alexa Fluor 488, Bioss, Shanghai, China; 1:100 dilution) in the dark for 60 min at room temperature. The nuclear staining with DAPI (Wuhan Servicebio Technology Co., Ltd.) was performed after the sections were washed three times with PBS buffer.

### 2.6 Image analysis

The images obtained in the experiments were analysed and processed using Image Pro 6.0. Data were statistically analysed using SPSS 18.0 software and expressed as mean ± standard deviation. The comparisons between two groups and among multiple groups were performed based on *t*‐test and the one‐way analysis of variance (anova) analyses, respectively, with the statistical significance determined by the *Q*‐test based on *α* = 0.05 and *p* < 0.05 or *p* < 0.01.

## 3 RESULTS

### 3.1 Behavioural observation of rats with KA‐induced status epilepticus

In 30–50 min after the amygdala injection of KA (i.e. about 30–50 min after the withdrawal of isoflurane), the rats began to wake up, showing increased breathing rate and grade I seizures such as chewing and nodding. In 2–3 h, the seizures reached grade V, for example, tonic–clonic seizures, fallings and other convulsive seizures. After the injection of diazepam, the frequency and severity of convulsive seizures were reduced with prolonged interval between seizures until they stopped. In 24 h after SE, the rats showed spontaneous seizures. During the interictal period, the rats showed increased excitability, agitation, irritability and significantly reduced food and water intakes. In 24–72 h, the spontaneous seizures were reduced, the duration of seizures was shortened to several or tens of seconds, the interval between seizures was prolonged, food and water intakes were reduced, and the body weight was significantly reduced. From 72 h to 1 week, most of the rats were recovered to normal state with normal food and water intakes as well as increased body weight, occasionally showing spontaneous seizures. In 1 week and thereafter, the spontaneous seizures were hardly observed.

### 3.2 Behavioural observation of epileptic rats with intranasal or oral administrations of levetiracetam

Both LND and LPO groups were given the drug once 30 min prior to the modelling surgery. The time interval between the end of the KA injection and the onset of grade I seizure manifestations observed when the rats woke up was defined as the latency period. The latency periods for LND, LPO and EP groups were 40–50 min, showing no significant difference. In 2–3 h after the KA injection, rats in LND, LPO and EP groups all showed grade V seizures lasting for 1 h until the injection of diazepam terminated the seizures. In 24 h after SE, the rats showed frequent spontaneous seizures, agitation, irritability, and reduced food and water intakes. The frequency of seizures and the degree of agitation were largely varied among individual rats with no evident patterns observed among the three groups. In 24–72 h, the spontaneous seizures were decreased and occasionally observed in LND and LPO groups, while no spontaneous seizures were observed even on the second day after SE. In the EP group, the spontaneous seizures were observed in most rats in 72 h. Due to the reduction of food and water intakes, the rats in these three experimental groups of rats experienced weight loss in 72 h after SE. However, compared with the EP group, the LND and LPO groups were recovered more quickly to normal habits of food and water intakes in 72 h. Therefore, the body weights of the EP group were significantly decreased than those of the LND and LPO groups. In 3 days to 1 week, the rats in the EP group were recovered to normal eating and drinking habits, showing increased body weights in all three experimental groups with the spontaneous seizures hardly observed.

### 3.3 Effects of levetiracetam on pathological changes of hippocampus in epileptic rats

**FIGURE 2:**
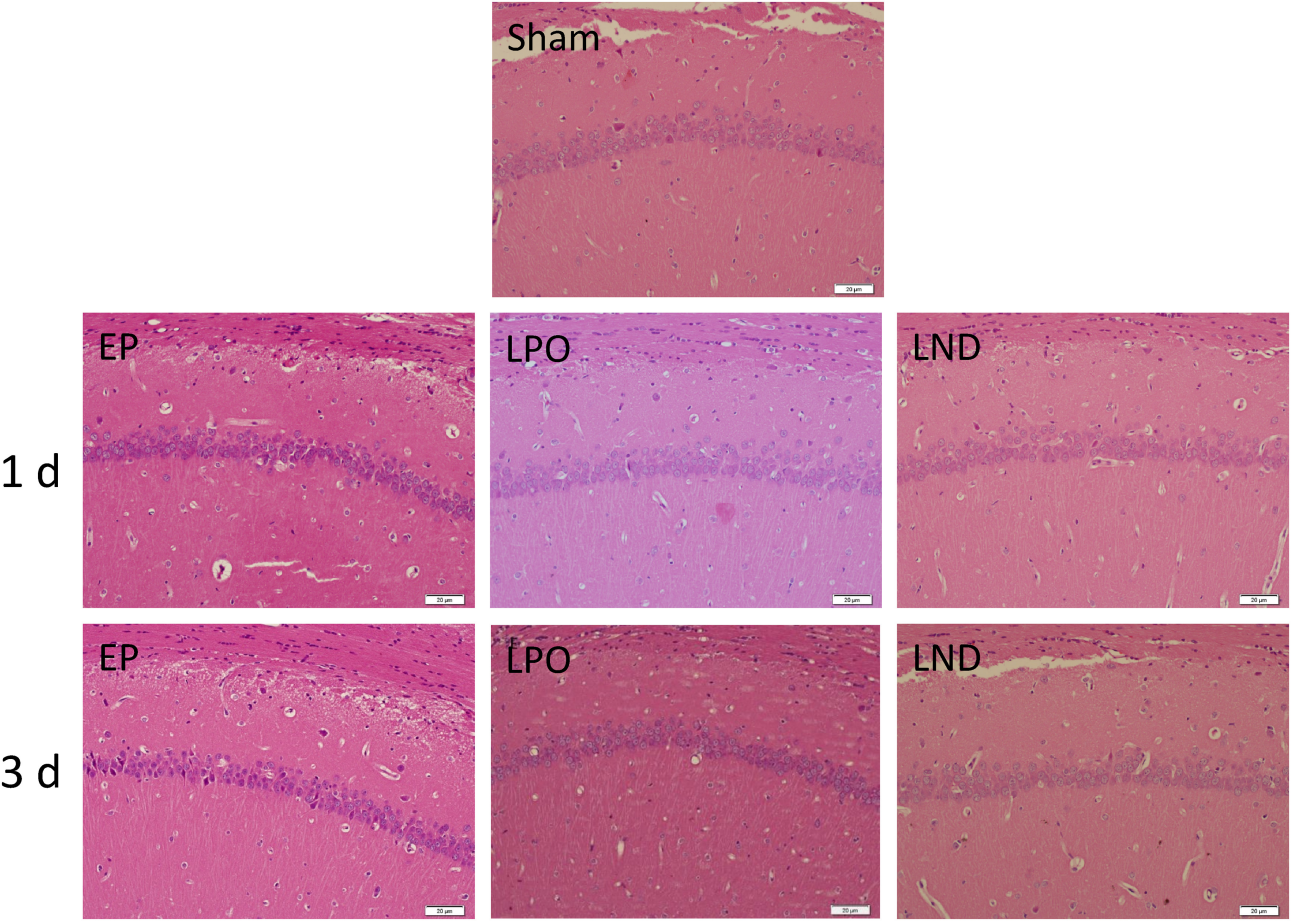
Haematoxylin and eosin staining of the CA1 area of hippocampus in rats of Sham group and of EP, LPO and LND groups 1 day and 3 days after status epilepticus.

**FIGURE 3:**
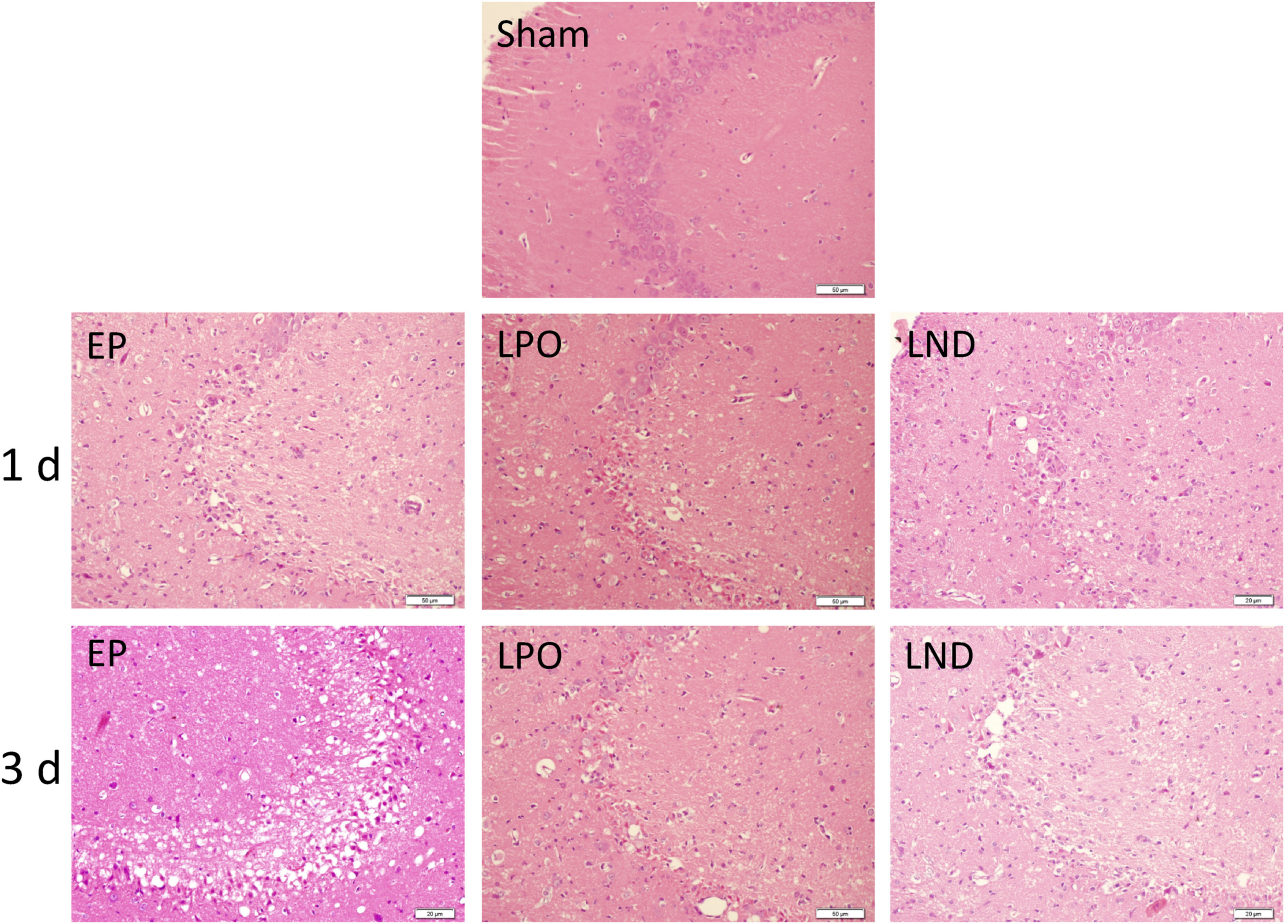
Haematoxylin and eosin staining of the CA3 area of hippocampus in rats of the Sham group and of the EP, LPO and LND groups 1 day and 3 days after status epilepticus.

The pathological changes in the CA1 (Figure 2) and CA3 (Figure 3) areas of hippocampus in the rats after the epileptic seizures were observed based on the haematoxylin and eosin staining. In the CA1 area, the pyramidal neurons in the Sham group showed normal morphology with intact structure and largely normal arrangement. On Day 1 and 3 after SE, the pyramidal cell layers in the EP, LPO and LND groups were largely neatly arranged, showed normal morphology of neurons and regular shape of the nucleus. Only slight damages were observed, that is, a small amount of neurons shed or disappeared, leaving cell debris and neurons with evident neuronal apoptosis signs such as pyknosis, fragmentation, dissolution and disappearance of nucleoli.

In the CA3 area, the Sham group showed that the pyramidal neurons were normal in shape, neatly arranged and intact in structure. In 1 day after SE, a large number of neurons of the EP group were shed and disappeared, leaving only cell debris, with evident neuronal apoptosis signs such as pyknosis, fragmentation, dissolution and disappearance of nucleoli. Similar pathological changes were observed in the LPO and LND groups to those of the EP group. The difference was that the numbers of necrotic cells and neurons lost in LPO and LND groups were significantly lower than that in the EP group. On Day 3, the numbers of neurons lost in the EP, LPO and LND groups were increased compared with those on Day 1. The morphology of pyramidal cell layers was largely lost in the EP group with the remaining neurons showing disordered arrangement. The morphology of the pyramidal cell layers of the LPO and LND groups was maintained with significantly less damage to the tissue than that in the EP group, while no significant difference was observed in the tissue damage between LPO and LND groups.

### 3.4 Effects of intranasal administration of levetiracetam on the expression of SV2A in epileptic rats

**FIGURE 4:**
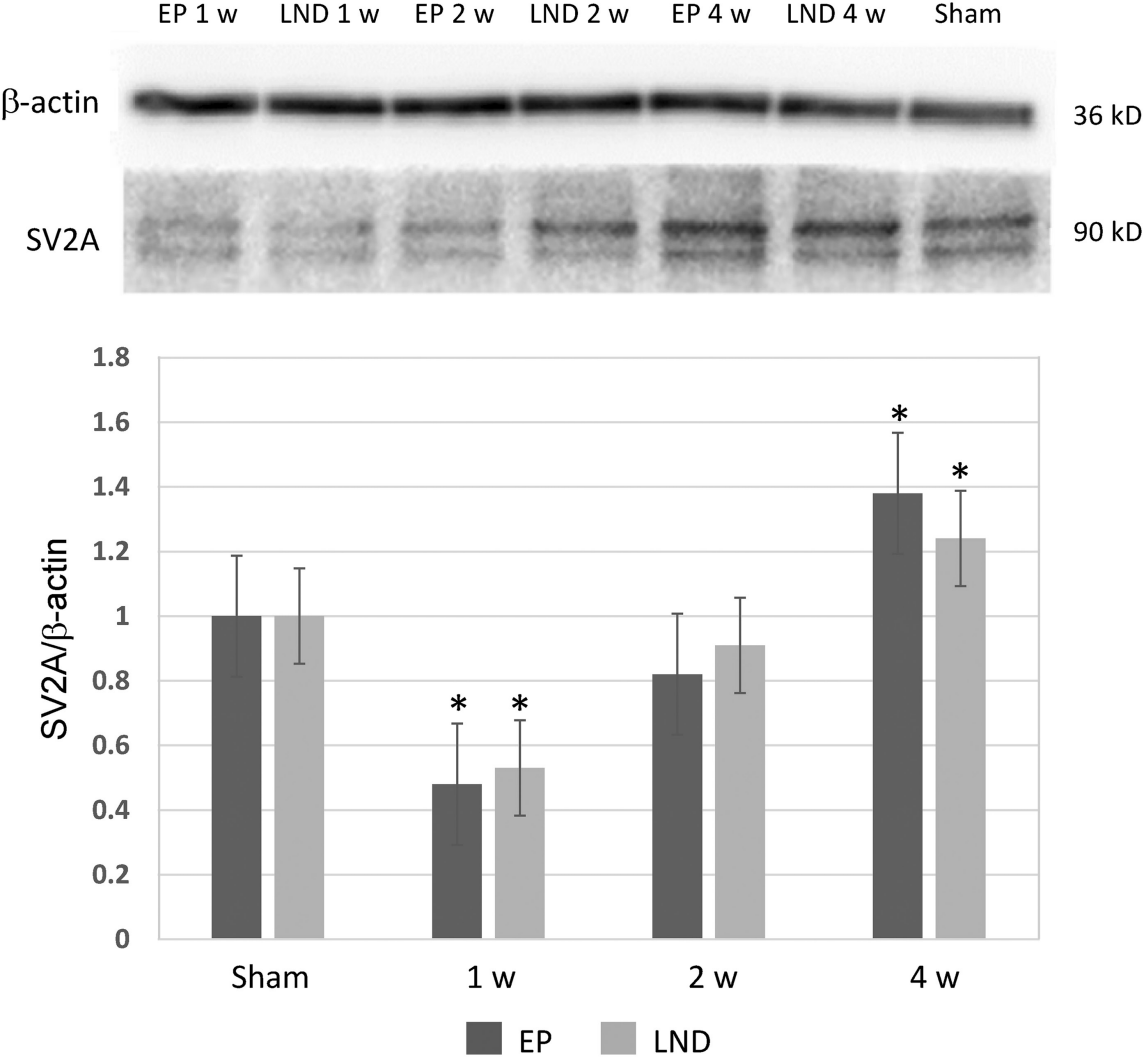
Western blotting analysis of the expression of SV2A in hippocampus tissue of rats in EP and LND groups in 1, 2 and 4 weeks after status epilepticus. Symbol ‘*’ indicates the significant difference based on *t*‐test with *p* < 0.05 in comparison with the Sham group.

The expression levels of SV2A after SE were detected by western blotting analysis in the hippocampus of rats in Sham, EP and LND groups (Figure 4). The expression of SV2A in the EP group showed largely the same pattern as that in the LND group. The total expression of SV2A was significantly lower than that of the Sham group in 1 week after SE and was increased in 2 weeks to the level similar to that of the Sham group. The expression level in 4 weeks was higher than that in 2 weeks and was significantly higher than that in the Sham group. No significant difference was observed in the expression of SV2A at each time point between the EP and LND groups, while the pairwise comparisons between adjacent time points within each of these two groups revealed statistically different expressions of SV2A in the rat hippocampus (*p* < 0.05).

**FIGURE 5:**
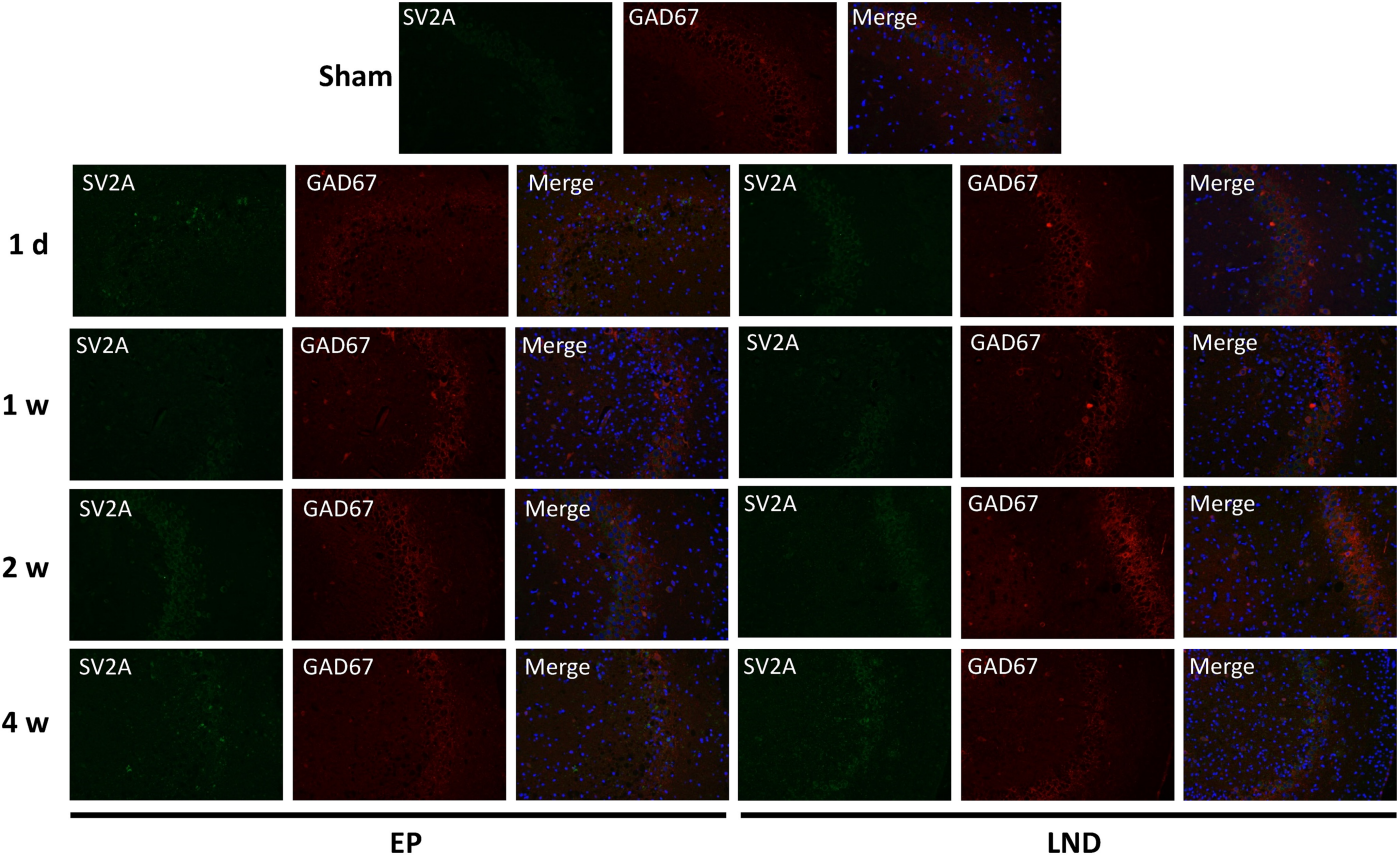
Immunofluorescence staining of GAD67 and SV2A in the CA3 region of hippocampus in rats of Sham group and of EP and LND groups at 1 day, 1 week, 2 weeks and 4 weeks after status epilepticus.

**FIGURE 6:**
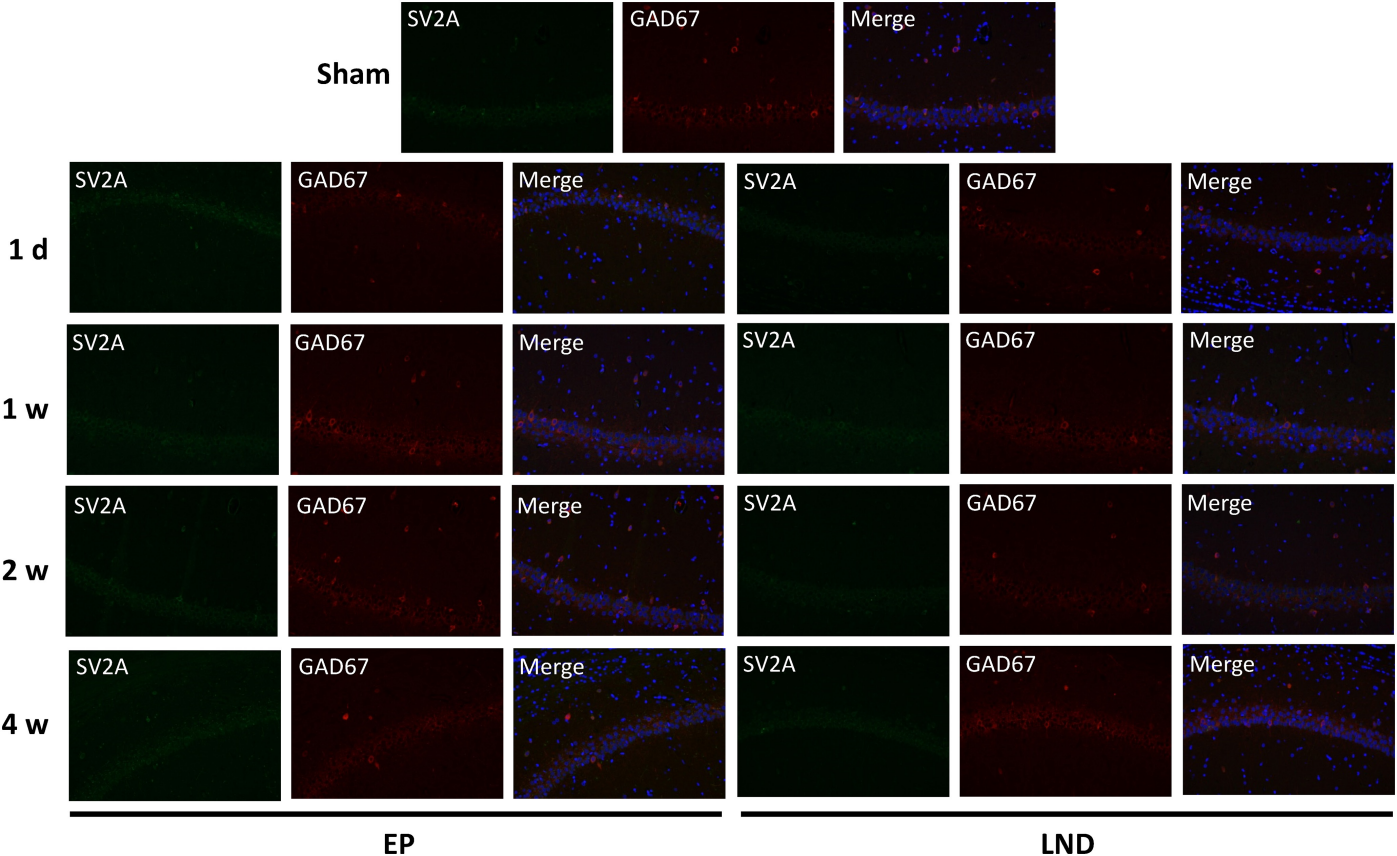
Immunofluorescence staining of GAD67 and SV2A in the CA1 region of hippocampus in rats of Sham group and of EP and LND groups at 1 day, 1 weeks, 2 weeks and 4 weeks after status epilepticus.

The spatial and temporal distributions of SV2A were detected by immunofluorescence staining of the CA3 (Figure 5) and CA1 (Figure 6) regions of hippocampus in rats of Sham, EP and LND groups (Table 1). The results showed that the high expression of SV2A was observed in the lamina pellucida, which mainly received the mossy fibres from dentate granule neurons. The SV2A expression was also observed around the pyramidal neurons in the pyramidal cell layers. The SV2A was co‐expressed with GAD67 in the pyramidal cell layers, suggesting the specific expression of SV2A in GABAergic pyramidal neurons but not in the lamina pellucida.

**TABLE 1:** Immunofluorescence staining of SV2A in CA3 and CA1 areas in four groups of rats.

Group	1 day	1 week	2 weeks	4 weeks
Area CA3				
Sham	127.07 ± 7.67			
EP	121.28 ± 13.83	71.31 ± 15.59*	134.41 ± 11.65	185.02 ± 9.44**
LND	143.15 ± 15.13	68.55 ± 17.34*	107.29 ± 7.30	184.26 ± 6.57**
Area CA1				
Sham	124.96 ± 21.25			
EP	128.12 ± 4.90	146.04 ± 22.39	132.83 ± 17.75	147.09 ± 3.55
LND	149.26 ± 7.11	140.79 ± 7.23	136.82 ± 6.03	141.10 ± 5.64

*Note*: Data are presented as mean ± standard deviation (*n* = 6) with significant difference compared with Sham group determined by *p* < 0.05 (*) and *p* < 0.01 (**), respectively.

Both SV2A and GAD67 were co‐expressed in the CA3 region of hippocampus in rats (Figure 5). The expression level of SV2A in the EP group was not significantly changed in 24 h after SE but was significantly lower than that in the Sham group in 1 week. In 2 weeks, the expression level was increased to the level similar to that of the Sham group and was significantly increased in 4 weeks in comparison to the Sham group. In the LND group, the expressions of SV2A were largely the same as those of the EP group. No significant differences were observed in the expressions of SV2A in the CA3 region at each time point between the EP and LND groups. From 24 h to 4 weeks after SE, the expressions of SV2A in the CA1 region in both EP and LND groups were not significantly changed with no significant difference observed at each time point between the EP and LND groups (Figure 6).

## 4 DISCUSSION

### 4.1 Pathological changes of hippocampus in rats after SE

In our study, significant tissue damage was revealed in the CA3 region of the hippocampus on the surgery side in the rat intra‐amygdala KA model of SE induced by KA, while the CA1 area on the same side of CA3 showed only mild but clear sporadic neuronal death (Figures 2 and 3). These results were consistent with those previously reported.^25^ For example, studies have shown that the KA receptors are highly expressed in both the presynaptic and post‐synaptic regions of the hippocampus.^26^

Previous studies showed that the hippocampal tissue damage reached the peak in 3 days after SE.^27^, ^28^ For example, in 1 week after SE, the morphology of the pyramidal cell layers disappeared and the proliferation of glial cells and new capillaries began to appear.^28^ In 2 weeks, the morphology of the pyramidal neuron layers in the CA3 area was the same as that in 1 week after SE, while the arrangement of neurons was disordered, with a large number of glial hyperplasia foci and new capillary growth observed.^28^ In 4 weeks, the pyramidal neuron layer in CA3 region was significantly more regular than that in 2 weeks, the number of pyramidal neurons was increased, and the evident interneuron glial hypertrophy and scarring were observed.^28^ These results were consistent with the findings revealed in our study (Figure 3), showing that in the CA3 area, the significantly increased damages were observed on Day 3 than Day 1 in all 3 groups (EP, LND and LPO); compared with the Sham group, all these 3 groups showed neurons shedding and disappearing in the CA3 area with evident neuronal apoptosis signs on Day 3, while obviously milder signs were observed on Day 1, suggesting that the damage of the CA3 area continued to increase in 3 days after SE.

It is well‐known that both neuronal loss and gliosis play important roles in the recurrence of epilepsy. For example, studies showed that the number of GABAergic inhibitory interneurons in the hippocampus and the CA3 area in chronic epilepsy were decreased, while the excitatory dentate gyrus granule cells were increased to promote the excitation‐inhibition balance, ultimately causing the epilepsy recurrence.^28^ These results were consistent with the findings revealed in the immunofluorescence staining analysis in our study (Figure 4), showing that both SV2A and GAD67 were co‐expressed in the CA3 area, suggesting the specific expression of SV2A in GABAergic pyramidal neurons but not in the lamina pellucida. Furthermore, the results of immunofluorescence staining showed that the expression level of SV2A was significantly decreased in 1 week after SE, suggesting a decline in the number and function of GABAergic neurons. However, the SV2A expression was increased in 2 weeks after SE, suggesting the mossy fibre sprouting and synaptic reconstruction of hippocampal.

### 4.2 Effects of nasal administration of levetiracetam

In our study, the rats were treated either orally or nasally with LEV of a therapeutic dose 30 min prior to the construction of SE models, and the LEV was continuously administered every 12 h thereafter. The behavioural observations of the rats after surgery showed that compared with the EP group, the spontaneous seizures of the rats treated with LEV either nasally or orally (i.e. both LND and LPO groups) were decreased rapidly and the time needed to recover to normal eating and drinking habits was shorter than that of the EP group. These results suggested that both intranasal and oral administrations of LEV showed antiepileptic effect after the epileptic seizures in rats. These findings were further verified by the results of the pathological changes in the hippocampus after SE (Figure 3). These results strongly indicated that the LEV administrated either orally or nasally entered into the brain tissue to play its protective role in the hippocampal neurons. Therefore, it was concluded that the intranasal administration was an effective way for LEV to enter the brain to play its antiepileptic and neuroprotective functions, showing the same therapeutic effects as those of the nasal administration of LEV.

To date, most of the investigations on nasal administration of AEDs are focused on the pharmacokinetics, while the studies on the efficacy of AEDs are sparse.^29^, ^30^, ^31^ Recent studies have used the intravenous administration of medicines to compare the effects with those of the nasal administration.^32^, ^33^ Our study was novel in its connection between the nasal administration of LEV and the LEV binding site in the brain tissue as well as the molecular mechanisms underlying the therapeutic effects of LEV in the treatment of epilepsy, revealing the similar efficacy of nasal and oral administrations of LEV. These consistent findings between the nasal and oral administrations of LEV were likely due to the time of administration selected in this study. In order to be more effectively and accurately observing the onset speed of LEV with nasal administration, it could be ideal to administrate LEV during SE. However, the behavioural and electro‐physiological observations would be interrupted due to the requirement of drug administration under anaesthesia. Future studies are necessary to improve these experimental designs in order to administrate drugs during SE.

### 4.3 Temporal and spatial distributions of SV2A and its interaction with levetiracetam in the rat hippocampus after SE

Many studies have revealed a positive correlation between the binding rate of SV2A with the LEV analogs and the efficacy of AEDs, suggesting that SV2A is the target of LEV.^34^, ^35^ Although the molecular mechanism underlying the effects of SV2A is currently unclear, it has been suggested that SV2A could regulate the synaptic vesicle exocytosis and play an important role in the homeostasis of synaptic vesicle components (e.g. ATP or Ca^2+^). Studies have shown that the disruption of gene encoding SV2A resulted in the decreased action potential‐dependent GABAergic neurotransmission in the hippocampal CA3 region.^19^ Moreover, van Vliet et al.^15^ reported that in the acute stage of epilepsy (1 day after SE), the expressions of SV2A in the molecular layer of the hippocampus and the hilar region of the dentate gyrus were decreased, but were still expressed in the nerve fibres in the hilum region and the dendrites of pyramidal neurons in the CA1 and CA3 regions. These results were consistent with the findings revealed by the immunofluorescence analysis in our study (Figures 5 and 6). The rapid reduction in SV2A could be explained by the neuronal degeneration after SE. For example, studies showed that in rats, the cell death occurred mainly during the latent period, that is, about 1 week after SE, corresponding to the decline in SV2A expression, in particular, in neurons projected to the molecular layer in the hilum area.^36^ From 2 to 4 weeks after SE, the expression of SV2A in the hippocampus were continuously increased, probably due to the formation of new axon collaterals and new synapses caused by the mossy fibre sprouting and synapse reconstruction in the chronic phase of epilepsy, resulting in the increase in the distribution area of the synaptic vesicles and increased SV2A expression. Our study revealed that the intranasal administration of LEV showed no significant effect on the expression of SV2A in the hippocampus of epileptic rats and the distribution of SV2A in each subdivision of the hippocampus (Figures 4, 5, 6), suggesting that the antiepileptic effects of LEV was not achieved by regulating the expression of SV2A. These results were consistent with those previously reported in human.^37^ Therefore, it was speculated that the drug resistance of LEV in the treatment of epilepsy was probably due to the reduced efficacy caused by the reduction of the drug binding sites. The similar effect of both nasal and oral administrations of LEV observed in our study was probably due to the decrease in the expression of SV2A as the LEV binding site or the relative saturation of the binding between LEV and SV2A. Both clinical and animal studies have shown that LEV initially showed strong inhibitory effect on epilepsy but the drug efficacy was declined during the chronic treatment in epileptic rats.^38^, ^39^ Further studies are needed to elucidate the effect of variations in the expression of SV2A on the neuronal excitability and efficacy of LEV in the treatment of epilepsy.

## AUTHOR CONTRIBUTIONS

**Hongmei Meng:** Project administration (lead); supervision (lead). **Weixuan Zhao:** Data curation (lead); formal analysis (lead); writing – original draft (lead); writing – review and editing (lead). **Yue Li:** Data curation (equal). **Huaiyu Sun:** Formal analysis (equal). **Wuqiong Zhang:** Validation (supporting). **Jiaai Li:** Conceptualization (supporting). **Ting Jiang:** Methodology (supporting). **Li Jiang:** Supervision (supporting).

## FUNDING INFORMATION

This work was support by the Department of Science and Technology, Jilin Province, China (Grant No. 20190304045YY to H.M.) and the Department of Finance, Jilin Province, China (Grant No. JLSWSRCZX2020‐0054 to H.M.).

## CONFLICT OF INTEREST STATEMENT

The authors declare no conflict of interest. The sponsors had no role in the design, execution, interpretation or writing of the study.

## Data Availability

Data are available from the corresponding author upon reasonable request
